# Complication rates and management in sentinel lymph node biopsy for endometrial cancer: a retrospective cohort study

**DOI:** 10.3389/fmed.2026.1774295

**Published:** 2026-02-11

**Authors:** Jing Yang, Ping Wu, Huimin Liu

**Affiliations:** Department of Gynecology, Hefei First People's Hospital, Hefei, China

**Keywords:** complications, endometrial cancer, lymphedema, patient-reported outcomes, pelvic lymph node dissection, sentinel lymph node biopsy

## Abstract

**Background:**

Systematic pelvic lymph node dissection (PLND) is the conventional staging procedure for early-stage endometrial cancer but is associated with substantial morbidity, particularly lower-limb lymphedema. Sentinel lymph node biopsy (SLNB) is a less invasive alternative, yet real-world evidence on complications and patient-reported quality of life (QoL) remains limited.

**Objective:**

To compare perioperative outcomes, postoperative complications, and QoL between SLNB and PLND in low- to intermediate-risk endometrial cancer, and to determine whether surgical approach is an independent risk factor for complications.

**Methods:**

We retrospectively analyzed 150 eligible patients with early-stage endometrial cancer treated at a gynecologic oncology center between January 2020 and December 2023, with ≥12 months of follow-up. Based on contemporaneously documented clinical decision-making and patient preference, patients were assigned to the SLNB group (*n* = 100) or the PLND group (*n* = 50). SLNB was performed using cervical indocyanine green injection with near-infrared fluorescence imaging; PLND comprised systematic pelvic lymphadenectomy. Outcomes included operative time, estimated blood loss, length of hospital stay, overall complications graded by Clavien–Dindo, 12-month lymphedema incidence, SLN mapping success rate, lymph node pathology (including ultrastaging), and QoL assessed by the EORTC QLQ-C30 preoperatively and at 3, 6, and 12 months. Univariate and multivariate logistic regression analyses were conducted to identify independent risk factors for postoperative complications.

**Results:**

Baseline characteristics (age, BMI, FIGO stage) were comparable between groups (all *p* > 0.05). Compared with PLND, SLNB was associated with shorter operative time (*p* < 0.001), lower blood loss (p < 0.001), and shorter postoperative hospital stay (*p* = 0.001). The patient-level SLN mapping success rate was 97.0%. Overall complication rates (*p* < 0.001) and 12-month lymphedema incidence (p < 0.001) were significantly lower in the SLNB group. Ultrastaging detected six additional cases of micrometastases or isolated tumor cells in the SLNB group (*p* = 0.016). Global health status scores were higher after SLNB at 3 months (*p* = 0.007) and 6 months (*p* = 0.041). In multivariate analysis adjusting for age, BMI, diabetes, and FIGO stage, PLND remained an independent risk factor for postoperative complications (aOR 4.732; 95% CI 2.029–11.034; *p* < 0.001).

**Conclusion:**

In low- to intermediate-risk early-stage endometrial cancer, SLNB provides effective staging with reduced surgical burden, fewer postoperative complications—particularly lymphedema—and earlier recovery of QoL compared with systematic PLND.

## 1 Introduction

Endometrial cancer is the most common gynecologic malignancy, with comprehensive and precise surgical staging forming the cornerstone of its management (1). Traditional staging involves systematic pelvic lymph node dissection (PLND) to evaluate nodal metastasis and guide adjuvant therapy (2). However, PLND, as an extensive lymphatic tissue resection, carries non-negligible risks of complications, particularly postoperative lower-limb lymphedema, which occurs in 20–40% of cases and severely impacts long-term quality of life (3, 4). In the current era of evidence-based precision and minimally invasive therapy, sentinel lymph node biopsy (SLNB) has been introduced into the surgical management of endometrial cancer (5). This technique uses tracer agents to identify the first lymph node draining the primary tumor region—the sentinel lymph node (SLN)—followed by ultrastaging pathological assessment, theoretically enabling accurate nodal evaluation while reducing surgical trauma (6, 7).

Multiple prospective and retrospective studies have confirmed that SLNB exhibits comparable nodal detection sensitivity to PLND in early-stage endometrial cancer while significantly reducing perioperative complications (8, 9). With the widespread adoption of cervical indocyanine green (ICG) injection combined with near-infrared fluorescence imaging, SLN detection and mapping success rates have improved markedly (10). However, existing evidence predominantly focuses on technical feasibility and oncological safety. Systematic prospective data on complication profiles specific to or reduced by SLNB—particularly long-term lymphedema incidence, severity, and impact on patient-reported outcomes (PROs)—remain limited (11, 12). Most studies are retrospective in design, susceptible to selection bias, and employ heterogeneous definitions and assessment criteria for complications, lacking standardized tools such as the Clavien-Dindo classification and validated PRO instruments like the EORTC QLQ-C30 (13, 14). Furthermore, variations in tracer selection, injection protocols, and ultrastaging procedures across institutions may contribute to result heterogeneity, obscuring the comprehensive benefits of SLNB in real-world clinical practice, especially its impact on quality of life (15).

To address these evidence gaps, this retrospective cohort study was conducted to systematically compare SLNB and PLND in patients with low- to intermediate-risk early-stage endometrial cancer within our institution. Surgical and pathological protocols were rigorously standardized, incorporating objective complication grading and validated quality of life measures. Perioperative outcomes, short- and intermediate-term complications (with emphasis on lymphedema), and dynamic recovery of patient-reported quality of life were prospectively collected and analyzed. Multivariate analysis controlled for potential confounding factors to clarify whether surgical technique itself independently influences complication risk, thereby providing higher-level clinical evidence on the safety, efficacy, and patient benefits of SLNB in endometrial cancer staging.

## 2 Materials and methods

### 2.1 General information

This single-center, retrospective cohort study evaluated the complication profiles and clinical management of SLNB versus systematic PLND in endometrial cancer patients. We reviewed the medical records of patients who underwent surgery at the Gynecologic Oncology Center of a university-affiliated hospital between January 2020 and December 2023. Data collection and analysis were performed in 2024. The study adhered to the principles of the Declaration of Helsinki and received approval from the institutional ethics review board.

A total of 162 patients were initially assessed for eligibility; after screening, 150 patients completed the study protocol and were included in the final analysis. The choice of nodal surgical procedure (SLNB or PLND) was made at the time of surgery based on contemporaneous clinical decision-making and patient preference, as documented in the medical records. This pragmatic allocation reflects real-world practice. This integrated assessment included preoperative pelvic MRI evaluation of myometrial invasion depth (<50% vs. ≥50%) and tumor size, combined with histological type (endometrioid vs. non-endometrioid) and grade (G1-3) from endometrial biopsy or dilation and curettage. Patients were considered low-to-intermediate risk if they had endometrioid histology, grade 1–2, and MRI suggesting <50% myometrial invasion or tumor size <2 cm, in the absence of clinical evidence of cervical involvement or extra-uterine disease [ref: e.g., ESGO/ESTRO/ESP guidelines or similar]. This pragmatic stratification informed the counseling regarding the suitability of SLNB versus PLND. Patients assessed as low to intermediate risk without evidence of nodal metastasis were thoroughly counseled on the risks and benefits of SLNB and PLND. Following detailed consultation, patients chose their surgical approach based on personal tolerance for surgical trauma and complication risks. Ultimately, 100 patients (66.7%) comprised the observation group (SLNB group), and 50 patients (33.3%) constituted the control group (systematic PLND group).

The sample size was calculated based on data from a pilot study, using the overall complication rate as the primary endpoint. With anticipated rates of 10% for the SLNB group and 30% for the PLND group, a two-sided test, an *α* level of 0.05, and a power (1–*β*) of 0.8, the PASS 15.0 software (NCSS, LLC) indicated a minimum requirement of 47 patients per group, totaling 94 patients. To account for potential incomplete data in the retrospective review, the sample size calculation incorporated an adjustment. This adjustment yielded a final target sample size of 150 patients to ensure adequate statistical power.

### 2.2 Inclusion and exclusion criteria

#### 2.2.1 Inclusion criteria

1. Female patients aged 18–75 years;
2. Preoperative pathological confirmation of endometrial cancer via endometrial biopsy or dilation and curettage;
3. Clinical stage I or II disease per FIGO 2023 criteria, based on gynecological examination, ultrasound, and pelvic MRI, indicating tumor confinement to the uterine corpus or cervical stromal invasion without evidence of extrauterine spread;
4. Planned standard tumor debulking surgery, including total hysterectomy and bilateral salpingo-oophorectomy;
5. mentally competent patients providing written informed consent after detailed explanation.

#### 2.2.2 Exclusion criteria

1. Preoperative imaging (e.g., chest CT, abdominal ultrasound, or MRI) suggesting distant metastasis (e.g., lung, liver, bone, or lymph nodes beyond the inguinal region);
2. Previous pelvic radiotherapy or any form of PLND for malignancy or other diseases;
3. Severe uncontrolled cardiovascular, pulmonary, hepatic, or renal dysfunction (e.g., NYHA class III or IV heart failure, end-stage liver disease, or chronic kidney disease requiring dialysis), deemed unfit for general anesthesia and surgery;
4. Pregnancy or lactation;
5. Cognitive impairment, psychiatric disorders, or any condition potentially compromising compliance with follow-up, as determined by the investigator.

### 2.3 Equipment and materials

Surgical and pathological procedures relied on standardized medical equipment to ensure technical consistency and result reliability. As this was an observational study comparing two defined surgical techniques, the operating surgeons were necessarily aware of the study objectives and the allocated nodal procedure (SLNB or PLND). Blinding was not feasible.

1. Surgical Operating System: The surgical approach (total laparoscopic hysterectomy or laparotomy) was selected based on patient anatomy, surgeon judgment, and equipment availability. The distribution of approaches is presented in the results.
2. SLN Tracer and Imaging System: Indocyanine green was used for lymphatic mapping. Sterile lyophilized ICG powder (25 mg) was reconstituted with injectable water to a concentration of 2.5 mg/mL. The Pinpoint near-infrared fluorescence imaging system, integrated with the laparoscopy platform, enabled real-time switching between white light and fluorescence modes. Upon tissue injection, ICG is absorbed by lymphatics and emits fluorescence under near-infrared excitation, delineating draining lymphatic vessels and accumulating SLNs.
3. Pathological Processing and Diagnostic Equipment: Resected lymph node specimens were promptly delivered fresh to the pathology department. Conventional pathological examination involved paraffin-embedded sectioning using a Leica RM2235 rotary microtome. Nodes negative on routine hematoxylin and eosin staining underwent further ultrastaging with the Leica BOND-III automated immunohistochemistry stainer, using broad-spectrum cytokeratin antibodies to detect micrometastases or isolated tumor cells missed by conventional methods.

### 2.4 Study methods

1. Study Design Framework: This retrospective study analyzed two parallel cohorts of patients who, in a real-world setting, underwent different nodal management strategies (SLNB or systematic PLND). All patients underwent radical hysterectomy and bilateral salpingo-oophorectomy, the standard surgical foundation for endometrial cancer.
2. Standardized Surgical Protocol: To minimize bias from inter-operator variability, all surgeries were performed by a dedicated team led by two senior surgeons, each with over 15 years of experience in gynecologic oncology.

Protocol for the Observation Group (SLNB): Under general anesthesia, patients were placed in the lithotomy position. After establishing pneumoperitoneum and inserting the laparoscope, a comprehensive abdominal exploration was performed. Subsequently, using a 1 mL syringe, 0.5 mL (approximately 1.25 mg) of the prepared ICG solution was injected slowly into the deep cervical stroma at the 3 and 9 o’clock positions, for a total dose not exceeding 5 mg. Immediately following injection, the laparoscope light source was switched to fluorescence mode to visualize and document fluorescent lymphatic channels draining from the cervix toward the pelvic sidewalls. The first or several fluorescent lymph nodes were defined as sentinel lymph nodes. These nodes were meticulously dissected and removed intact using an ultrasonic scalpel or bipolar electrocautery. In cases of failed unilateral mapping, a systematic lymphadenectomy was performed on that side.

Protocol for the Control Group (PLND): Patients in this group underwent systematic PLND. The surgical extent was standardized to include en bloc removal of the fibrofatty lymphatic tissue from the bilateral common iliac, external iliac, internal iliac, obturator, and deep inguinal nodal basins. Dissection aimed for anatomical completeness to achieve thorough regional nodal clearance.

1. Data Collection: Data were extracted retrospectively from the hospital’s electronic medical records (EMR) system using a standardized data collection form. Collected data included patient demographics, medical history, preoperative findings, operative time, estimated blood loss [using a combination of suction canister volume and the gravimetric method (weight gain of surgical gauzes, 1 g ≈ 1 mL)], and SLN mapping details (visualization status, number, and location of SLNs). Postoperative complications and follow-up data were extracted from clinical notes, discharge summaries, and records from scheduled follow-up clinic visits typically conducted at 1, 3, 6, and 12 months post-surgery. Assessments included physical examination and completion of quality of life questionnaires.
2. Ethics and Quality Control: The study protocol was reviewed and approved by the Institutional Ethics Review Board of [Please insert your hospital’s name here] Hospital [Approval No: (Please insert your approval number here)]. The requirement for informed consent was waived due to the retrospective nature of the study. Consent discussions, led jointly by the attending surgeon and a researcher, detailed the study’s purpose, procedures, potential risks, and benefits. All original medical records, pathology reports, and follow-up documentation were archived for audit purposes.
3. Postoperative lymphedema management protocols: For patients diagnosed with lymphedema in this study, a standardized management protocol was initiated. First-line therapy consisted of education on skin care, manual lymphatic drainage techniques taught by a physiotherapist, and compression garment use (20–30 mmHg knee-high stockings). Patients with moderate-to-severe or refractory symptoms were referred to a dedicated lymphedema clinic for complex decongestive therapy, which includes intensive multilayer bandaging, specialized massage, and tailored exercise programs.

### 2.5 Outcome measures

A combination of objective and patient-reported outcome measures was employed for a comprehensive comparison between the two nodal strategies.

1. Overall Complication Rate: All surgery-related adverse events occurring from the end of surgery until the last follow-up were recorded. Complications were categorized as short-term (≤30 days post-op) or long-term (>30 days post-op). Severity was uniformly classified using the internationally recognized Clavien-Dindo grading system. Analysis focused on complications directly related to the procedure, such as surgical site infection, lymphocyst infection/rupture, postoperative hemorrhage, deep vein thrombosis, and ileus, with particular attention to severe (Grade III, requiring intervention) or life-threatening (Grade IV) events.
2. Lower-Limb Lymphedema Incidence: This objective measure of long-term quality of life combined clinical assessment with patient report. Clinical Measurement: Following the Gynecologic Oncology Group (GOG) and common clinical trial criteria, bilateral lower limb circumference was measured at a fixed point 10 cm above the tibial tuberosity and at the point of maximal circumference using a non-elastic tape. Measurements were taken by two trained research nurses, and the average was recorded. Clinical lymphedema was defined as a persistent inter-limb difference of ≥2 cm at either measurement site, confirmed at two consecutive follow-up visits ≥4 weeks apart, in the absence of other causes (e.g., venous insufficiency). Limb dominance was recorded but not used to adjust the diagnostic threshold. Patient Report: Patients were asked about typical symptoms (heaviness, swelling, skin tightness, tightness of clothing/footwear).
3. Sentinel Lymph Node Mapping Success Rate: This evaluated the technical efficacy of SLNB. Success was defined at two levels: Patient-level success rate (proportion of patients with at least one SLN detected in either hemi-pelvis) and Bilateral/hemi-pelvic success rate (proportion with at least one SLN detected in both hemi-pelves). A bilateral success rate ≥85% was predefined as the benchmark for technical proficiency.
4. Operative Time: Precisely recorded as the total duration in minutes from skin incision to skin closure.
5. Intraoperative Blood Loss: Estimated as described above and recorded in milliliters.
6. Postoperative Hospital Stay: Recorded in days, calculated from the day of surgery (time zero) until the day the patient met standard medical discharge criteria (e.g., afebrile, ambulating independently, well-healed incision, resumed bowel function) and was formally discharged.
7. 30-Day Readmission Rate: Defined as the proportion of patients re-admitted within 30 days of discharge for any complication related to the index surgery (e.g., fever, wound infection, symptomatic lymphocyst, ileus).
8. Lymph Node Pathology: Nodal status (positive/negative) was recorded. Detailed analysis included the detection via ultrastaging of micrometastases (tumor cell clusters >0.2 mm but ≤2.0 mm in diameter) and isolated tumor cells (single cells or cell clusters ≤0.2 mm).
9. Patient-Reported Quality of Life (QoL): Assessed using the validated Chinese version of the European Organisation for Research and Treatment of Cancer Quality of Life Questionnaire-Core 30 (EORTC QLQ-C30) version 3.0. This 30-item instrument covers global health status, five functional scales (physical, role, emotional, cognitive, social), and multiple symptom scales. Raw scores were linearly transformed to a 0–100 scale per the manual. For functional and global health scales, higher scores indicate better QoL; for symptom scales, higher scores indicate greater symptom burden. Questionnaires were administered preoperatively (baseline) and at 3, 6, and 12 months postoperatively.

### 2.6 Statistical analysis

All analyses were performed using IBM SPSS Statistics software (Version 26.0). Continuous variables were tested for normality using the Shapiro–Wilk test. Normally distributed data are presented as mean ± standard deviation and compared using the independent samples t-test. Non-normally distributed data are presented as median (interquartile range) and compared using the Mann–Whitney U test. Categorical variables are presented as frequency (percentage) and compared using the Chi-square test or Fisher’s exact test (when expected frequencies were <5).

To assess the independent impact of nodal strategy on postoperative complications, logistic regression analysis was employed. Univariate logistic regression was first performed to calculate the unadjusted odds ratio (OR) and 95% confidence interval (CI) for the association between each potential predictor (including surgical group, age, BMI, FIGO stage, histologic grade, diabetes, etc.) and complication occurrence. Subsequently, a multivariate logistic regression model was constructed to evaluate the adjusted effect of surgical approach (with SLNB as the reference). To control for confounding, the model included variables with *p* < 0.10 in univariate analysis along with pre-specified variables of clinical importance (e.g., age, FIGO stage). Variables considered potential mediators on the causal pathway were intentionally excluded from the primary multivariate model to estimate the total effect of the surgical technique itself. Specifically, operative time and intraoperative blood loss are not confounders but are directly influenced by the extent of lymphatic dissection (SLNB vs. PLND) and are themselves likely causes of postoperative complications. Adjusting for these intermediates would attenuate the estimated total effect of the surgical strategy on complication risk. Therefore, they were excluded from the main adjustment model to answer the primary clinical question regarding the overall outcome difference between the two surgical approaches. The final multivariate model included surgical group, variables with *p* < 0.10 in univariate analysis (BMI, diabetes), and clinically relevant pre-specified covariates (age, FIGO stage, surgical approach, ASA class, and tumor size). Results are reported as adjusted odds ratio (aOR) with 95% CI.

For longitudinal comparison of QoL scores, repeated-measures ANOVA was used to evaluate time effect, group effect, and their interaction. Learning curve analysis for the SLN mapping technique was performed and visualized using a cumulative summation (CUSUM) chart. To further address potential confounding due to non-random allocation, a supplementary sensitivity analysis was performed using propensity score matching (PSM). A logistic regression model was used to generate propensity scores based on age, BMI, FIGO stage, histologic grade, and diabetes mellitus. One-to-one nearest-neighbor matching without replacement was performed with a caliper width of 0.2 standard deviations of the logit of the propensity score. Outcome comparisons were repeated in the matched cohort. All statistical tests were two-sided, with *p* < 0.05 considered statistically significant.

## 3 Results

A total of 162 patients met the preliminary screening criteria. Of these, 12 were excluded due to loss to follow-up (*n* = 8; 5 in the SLNB group, 3 in the PLND group) or withdrawal of informed consent (*n* = 4; 3 in the SLNB group, 1 in the PLND group). Consequently, 150 patients completed all study procedures and were included in the final analysis (SLNB group: *n* = 100; PLND group: *n* = 50). All patients successfully underwent the planned radical surgery with no intraoperative mortality or conversions to laparotomy.

### 3.1 Patient baseline characteristics

Key baseline characteristics, including age, body mass index (BMI), FIGO stage, histologic type and grade, and comorbidities, were compared between the two groups (Table 1). Statistical analysis revealed no significant differences in any baseline characteristic between the groups (all *p* > 0.05). This indicates that despite non-random allocation, the groups were well-balanced on key demographic and clinicopathological features likely to influence surgical outcomes and complication risk, providing a reliable foundation for subsequent comparisons.

**Table 1:** Comparison of baseline characteristics between SLNB and systematic PLND groups.

Characteristic	SLNB group (*n* = 100)	PLND group (*n* = 50)	Statistic	*p*-value
Age (years), mean ± SD	58.34 ± 9.27	59.81 ± 8.96	*t* = −0.943	0.347
BMI (kg/m^2^), mean ± SD	26.45 ± 4.18	27.11 ± 4.33	*t* = −0.908	0.366
Menopausal status, *n* (%)			*χ*^2^ = 0.127	0.722
Premenopausal	23 (23.00)	10 (20.00)		
Postmenopausal	77 (77.00)	40 (80.00)		
FIGO stage (2023), *n* (%)			χ^2^ = 0.694	0.405
Stage I	85 (85.00)	44 (88.00)		
Stage II	15 (15.00)	6 (12.00)		
Histologic type, *n* (%)			Fisher’s Exact	0.506
Endometrioid	93 (93.00)	48 (96.00)		
Non-endometrioid	7 (7.00)	2 (4.00)		
Histologic grade, *n* (%)			*χ*^2^ = 0.948	0.623
G1	52 (52.00)	29 (58.00)		
G2	36 (36.00)	15 (30.00)		
G3	12 (12.00)	6 (12.00)		
Hypertension, *n* (%)	41 (41.00)	23 (46.00)	*χ*^2^ = 0.366	0.545
Diabetes mellitus, *n* (%)	19 (19.00)	12 (24.00)	*χ*^2^ = 0.523	0.469
Preoperative albumin (g/L), mean ± SD	39.87 ± 3.25	40.12 ± 3.41	*t* = −0.438	0.662
Preoperative anemia, *n* (%)	18 (18.00)	11 (22.00)	*χ*^2^ = 0.355	0.551
ASA Class, *n* (%)			*χ*^2^ = 0.213	0.899
I-II	88 (88.00)	43 (86.00)		
III	12 (12.00)	7 (14.00)		
Surgical approach, *n* (%)			*χ*^2^ = 0.048	0.826
Minimally invasive	94 (94.00)	48 (96.00)		
Open	6 (6.00)	2 (4.00)		
Preoperative CA125 (U/mL), M (IQR)	21.50 (15.80–32.25)	23.10 (16.60–35.40)	*Z* = −0.672	0.501
Tumor size (cm), mean ± SD	2.68 ± 1.24	2.81 ± 1.31	*t* = −0.587	0.558
Myometrial invasion, *n* (%)			*χ*^2^ = 0.023	0.879
< 1/2	71 (71.00)	35 (70.00)		
≥ 1/2	29 (29.00)	15 (30.00)		

### 3.2 Surgical and pathological outcomes

The patient-level sentinel lymph node mapping success rate was 97.00%, with a bilateral detection rate of 88.00%. Regarding surgical parameters, the SLNB group had significantly shorter operative time and lower estimated intraoperative blood loss compared to the PLND group (*t* = −8.632, *p* < 0.001; *Z* = −5.874, p < 0.001). The median postoperative hospital stay was also shorter in the SLNB group (*Z* = −3.214, *p* = 0.001). In terms of nodal pathology, the overall node-positive rate did not differ significantly between groups (*χ*^2^ = 0.021, *p* = 0.886). However, ultrastaging identified an additional 6 cases with low-volume metastasis (3 micrometastases, 3 isolated tumor cells) exclusively in the SLNB group, significantly enhancing the detection of such metastases (*χ*^2^ = 5.828, *p* = 0.016) (Table 2).

**Table 2:** Comparison of surgical parameters and lymph node pathology between groups.

Outcome	SLNB group (*n* = 100)	PLND group (*n* = 50)	Statistic	*p*-value
SLN mapping results				
Patient-level success, *n* (%)	97 (97.00)	–	–	–
Bilateral detection, *n* (%)	88 (88.00)	–	–	–
Surgical parameters				
Operative time (min), mean ± SD	152.37 ± 28.46	198.15 ± 35.72	*t* = −8.632	<0.001
Blood loss (mL), M (IQR)	80.00 (50.00–120.00)	150.00 (100.00–220.00)	*Z* = −5.874	<0.001
Postop. hospital stay (days), M (IQR)	5.00 (4.00–6.00)	6.00 (5.00–8.00)	*Z* = −3.214	0.001
Pathology results				
Node-positive, *n* (%)	14 (14.00)	6 (12.00)	*χ*^2^ = 0.021	0.886
Positive by conventional H&E	8 (8.00)	6 (12.00)		
Positive only by ultrastaging (ITC/Micromets)	6 (6.00)	0 (0.00)	*χ*^2^ = 5.828	0.016

### 3.3 Complication profile

**Figure 1:**
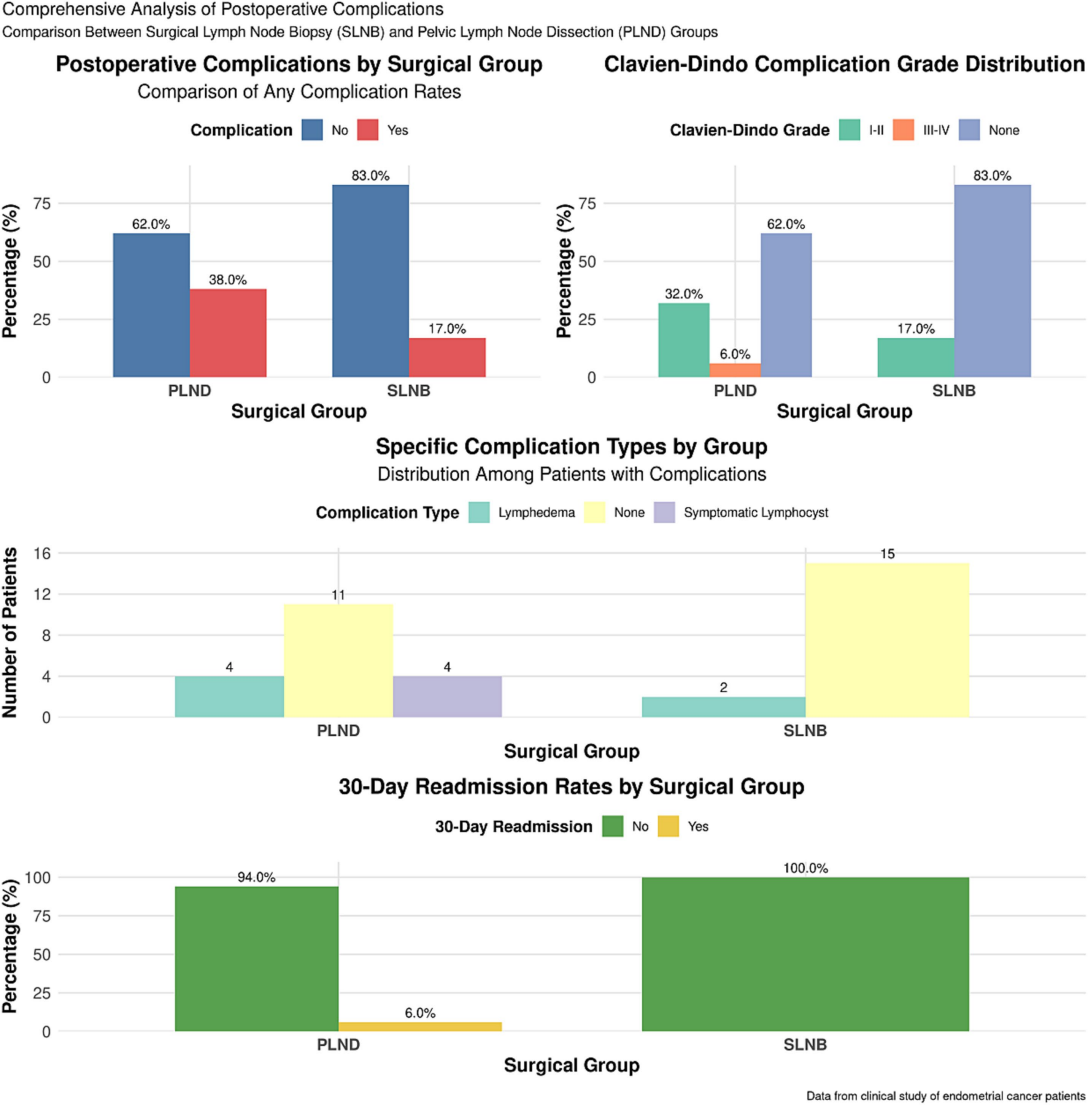
Comparison of postoperative complications between groups.

Analysis of overall postoperative complications revealed a significantly lower rate in the SLNB group compared to the PLND group (16.00% vs. 46.00%, χ^2^ = 15.752, *p* < 0.001). Regarding severe complications, no Clavien-Dindo grade III-IV events occurred in the SLNB group, whereas 4 such events (8.00%) occurred in the PLND group (Fisher’s exact test, *p* = 0.004). The most common complication in the SLNB group was symptomatic lymphocyst (4.00%), while lymphedema (26.00%) and symptomatic lymphocyst (12.00%) predominated in the PLND group. Notably, at 12 months postoperatively, the incidence of clinically diagnosed lower-limb lymphedema was significantly lower in the SLNB group (5.00% vs. 26.00%, χ^2^ = 14.060, *p* < 0.001). The 30-day readmission rate also trended lower in the SLNB group (2.00% vs. 8.00%), though this difference was not statistically significant (Fisher’s exact test, *p* = 0.106)(Table 3 and Figure 1). Regarding the impact on adjuvant therapy, postoperative complications led to a minor delay (2–3 weeks) in the initiation of planned vaginal brachytherapy for 2 out of the 23 patients (8.7%) with complications in the PLND group. No treatment delays occurred in the SLNB group.

**Table 3:** Comparison of postoperative complications between groups [*n* (%)].

Complication	SLNB group (*n* = 100)	PLND group (*n* = 50)	Statistic	*P*-value
Any complication	16 (16.00)	23 (46.00)	*χ*^2^ = 15.752	<0.001
By Clavien-Dindo grade				
Grade I-II	16 (16.00)	19 (38.00)		
Grade III-IV	0 (0.00)	4 (8.00)	Fisher’s Exact	0.004
Specific complications				
Lymphedema (at 12 months)	5 (5.00)	13 (26.00)	*χ*^2^ = 14.060	<0.001
Symptomatic lymphocyst	4 (4.00)	6 (12.00)	*χ*^2^ = 3.394	0.065
Surgical site infection	3 (3.00)	2 (4.00)	Fisher’s Exact	0.665
Deep vein thrombosis	2 (2.00)	1 (2.00)	Fisher’s Exact	1.000
Ileus	1 (1.00)	2 (4.00)	Fisher’s Exact	0.246
Postop. Hemorrhage (requiring intervention)	1 (1.00)	0 (0.00)	Fisher’s Exact	1.000
30-day readmission	2 (2.00)	4 (8.00)	Fisher’s Exact	0.106

### 3.4 Patient-reported quality of life

**Figure 2:**
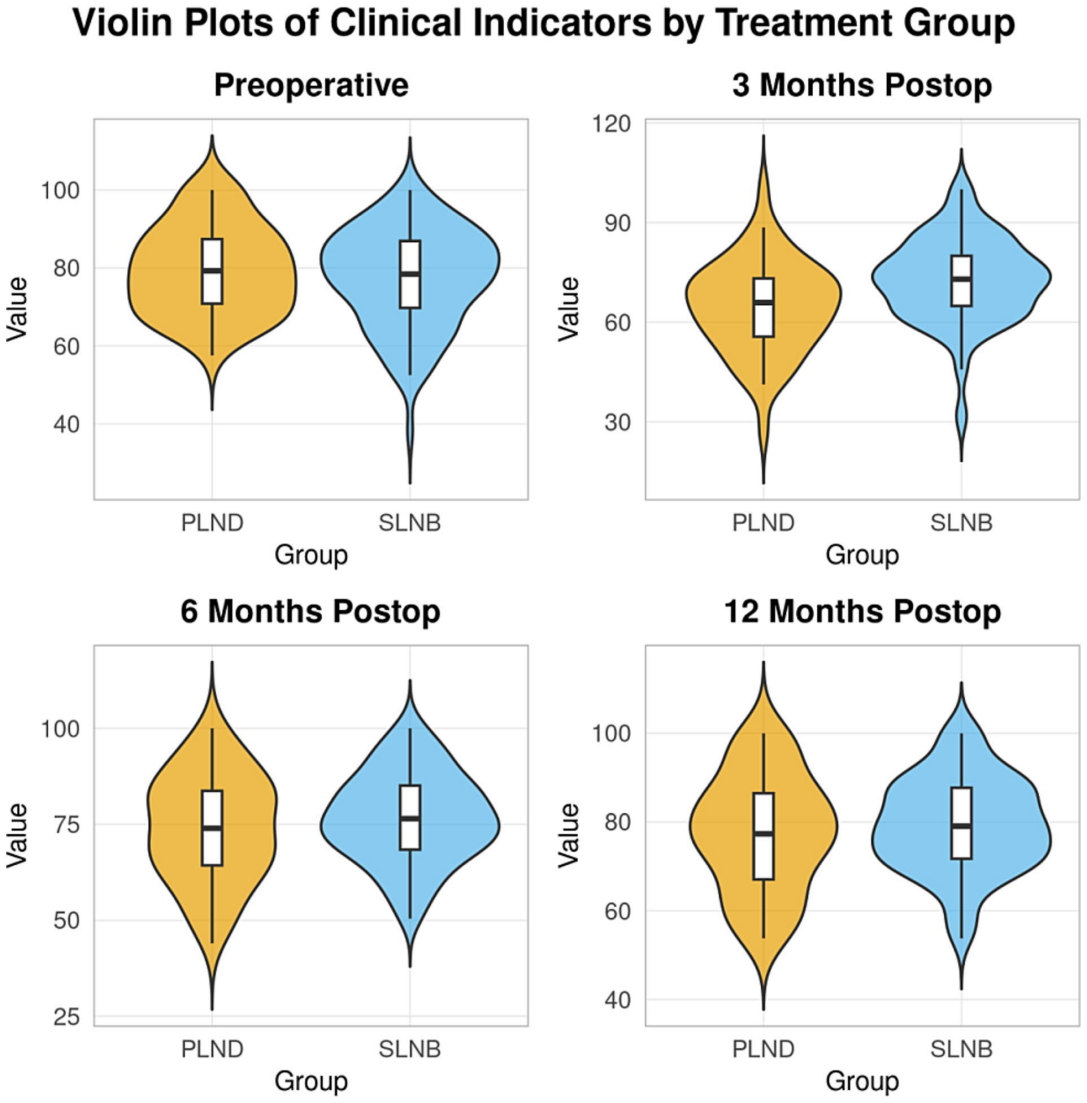
Comparison of EORTC QLQ-C30 global health status scores between groups.

Quality of life was assessed using the EORTC QLQ-C30 questionnaire. No significant difference was observed in preoperative baseline global health status scores between groups (*t* = −0.285, *p* = 0.776). Scores declined from baseline in both groups postoperatively before gradually recovering. Repeated-measures ANOVA revealed a significant interaction effect between time and group on global health status scores (*F* = 3.892, *p* = 0.010). Post-hoc pairwise comparisons showed that the SLNB group had significantly higher scores than the PLND group at 3 and 6 months postoperatively (*t* = 2.714, *p* = 0.007; *t* = 2.058, *p* = 0.041). By 12 months, the difference was no longer statistically significant (*t* = 1.573, *p* = 0.118) (Table 4 and Figure 2).

**Table 4:** Comparison of EORTC QLQ-C30 global health status scores between groups (mean ± SD).

Time point	SLNB group (*n* = 100)	PLND group (*n* = 50)	*t*-value	*P*-value
Preoperative (Baseline)	78.65 ± 12.34	79.27 ± 11.86	−0.285	0.776
3 Months postop.	70.42 ± 13.58	63.89 ± 14.25	2.714	0.007
6 Months postop.	76.18 ± 12.41	72.15 ± 13.67	2.058	0.041
12 Months postop.	80.33 ± 11.72	77.81 ± 12.93	1.573	0.118

Repeated-measures ANOVA showed a significant time-by-group interaction (*F* = 3.892, *P* = 0.010).

### 3.5 Analysis of sentinel lymph node Ultrastaging results

Ultrastaging was performed on a total of 352 sentinel lymph nodes from the 97 patients with successful SLN mapping. Conventional hematoxylin and eosin (H&E) staining identified 8 node-positive patients (involving 14 positive nodes). Immunohistochemical ultrastaging identified an additional 6 patients with low-volume metastasis: 3 with micrometastases (involving 4 nodes) and 3 with isolated tumor cells only (involving 3 nodes). This increased the final node-positive rate in the SLNB group from 8.25 to 14.43%. All metastases detected by ultrastaging were confined to sentinel nodes, with no metastases found in non-sentinel nodes. Ultrastaging increased the detection of low-volume metastasis by 75.00% (6/8) (Table 5).

**Table 5:** Details of ultrastaging pathology in the SLNB group (*n* = 97 patients with successful mapping).

Pathologic finding	Number of patients (*n*)	Percentage (%)	Number of involved nodes
Positive by Conventional H&E	8	8.25	14
Positive ONLY BY ultrastaging (ITC/Micromets)	6	6.19	7
Micrometastasis	3	3.09	4
Isolated tumor cells (ITC)	3	3.09	3
Total positive patients	14	14.43	21
Pathologically node-negative	83	85.57	–

### 3.6 Univariate analysis of factors associated with postoperative complications

Univariate logistic regression analysis was performed with the occurrence of any postoperative complication as the dependent variable (Table 6). Surgical group (PLND vs. SLNB), BMI, operative time, intraoperative blood loss, and presence of diabetes mellitus were significantly associated with complication occurrence (*p* < 0.10). Other variables, including age and FIGO stage (II vs. I), showed no significant association.

**Table 6:** Univariate logistic regression analysis of independent factors for postoperative complications.

Factor	B	SE	Wald	*P*-value	OR	95% CI
Surgical group (PLND vs. SLNB)	1.624	0.425	14.593	<0.001	5.074	2.204–11.684
BMI (per 1 kg/m^2^ increase)	0.131	0.059	4.932	0.026	1.140	1.016–1.279
Operative time (per 10 min increase)	0.108	0.045	5.760	0.016	1.114	1.020–1.217
Blood loss (per 50 mL increase)	0.243	0.120	4.101	0.043	1.275	1.008–1.613
Diabetes mellitus (Yes vs. No)	0.865	0.481	3.236	0.072	2.375	0.925–6.098
Age (per 5-year increase)	−0.018	0.110	0.027	0.870	0.982	0.791–1.219
FIGO stage (II vs. I)	0.511	0.506	1.020	0.312	1.667	0.619–4.491
Histologic grade (G2/G3 vs. G1)	0.288	0.383	0.566	0.452	1.334	0.629–2.828
Hypertension (Yes vs. No)	0.256	0.379	0.456	0.500	1.292	0.614–2.718
Constant	−3.825	0.925	17.106	<0.001	0.022	–

### 3.7 Multivariate analysis of independent factors for postoperative complications

**Figure 3:**
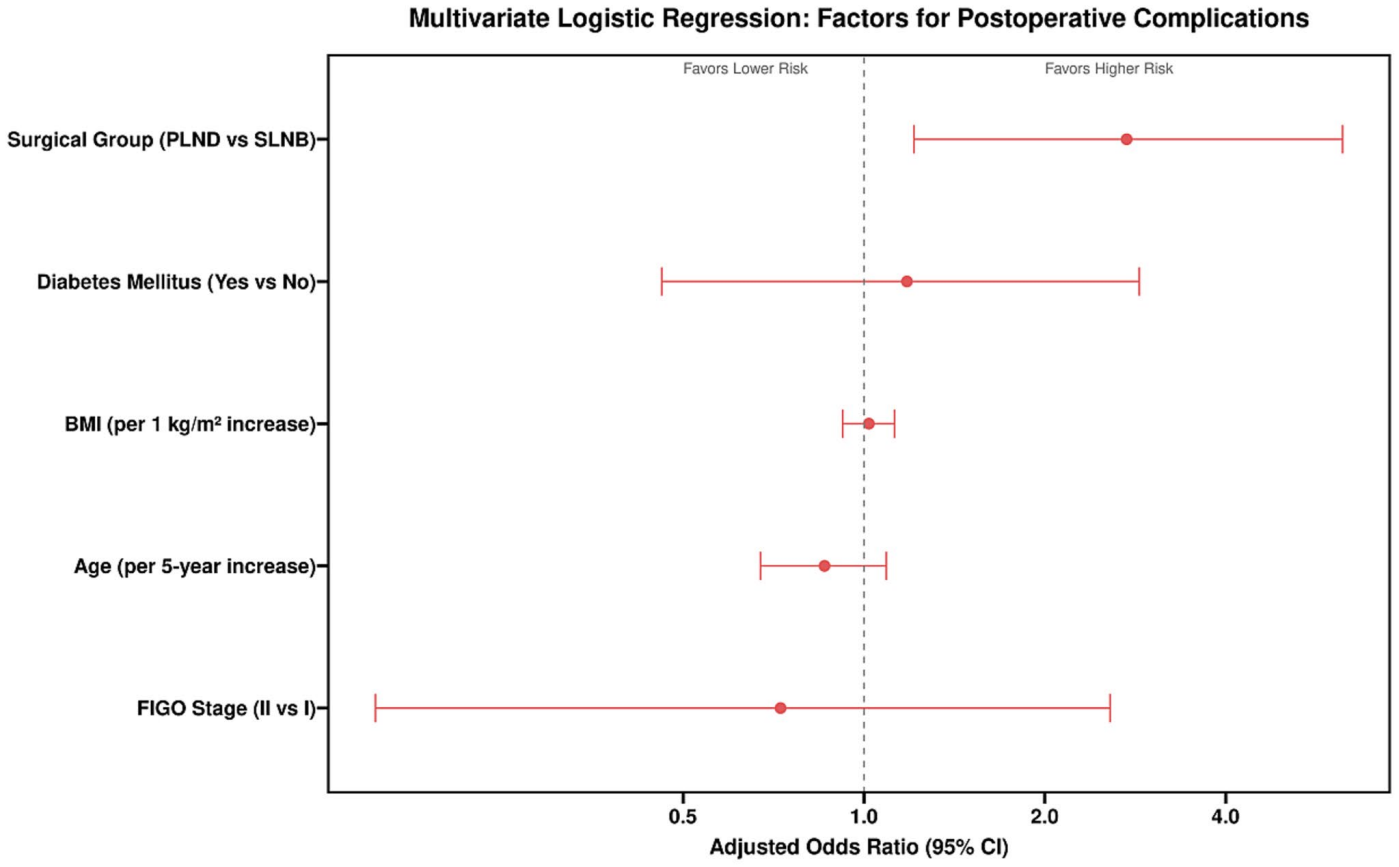
Multivariate logistic regression analysis of independent factors for postoperative complications.

**Figure 4:**
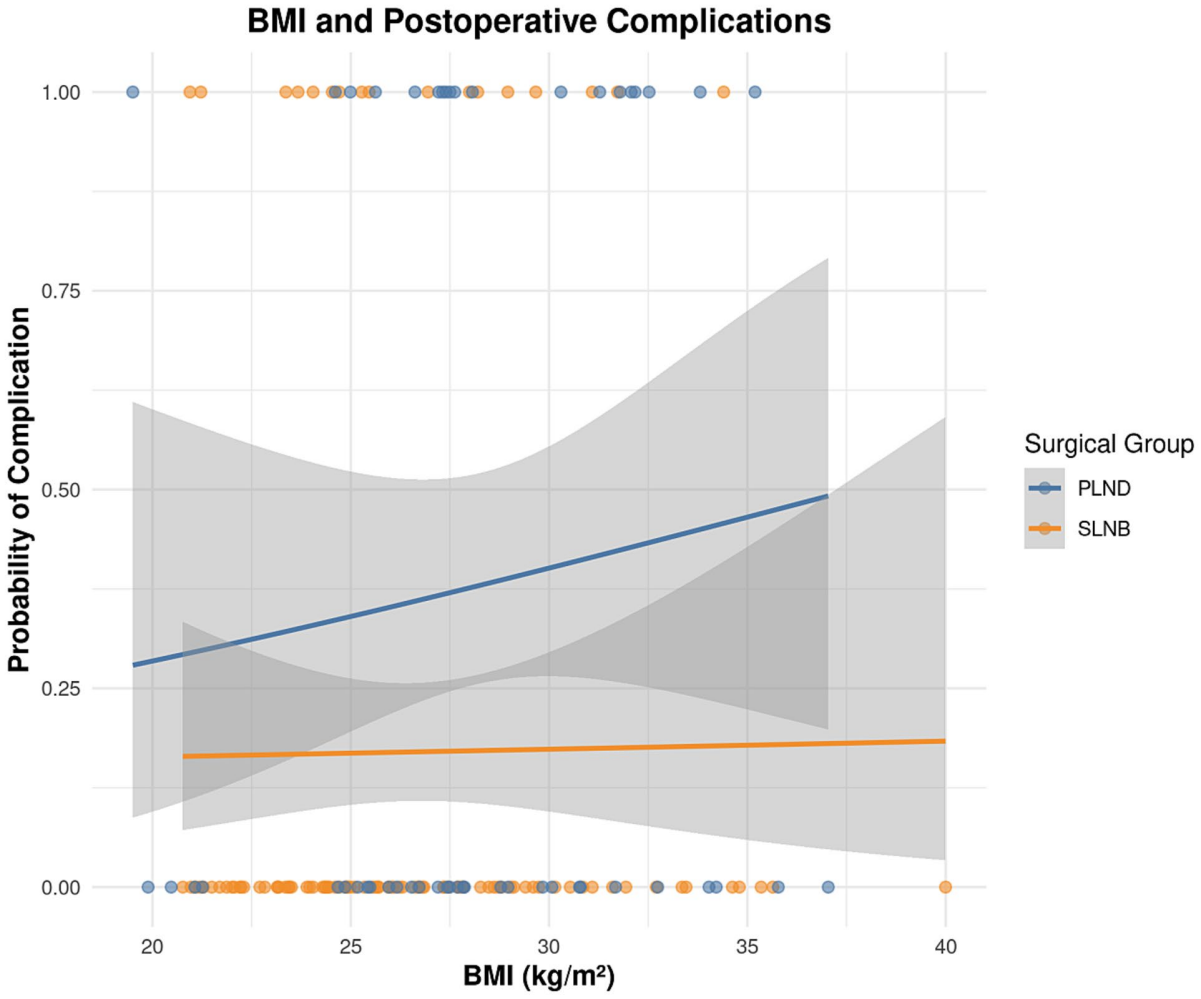
BMI and postoperative complications.

Variables with p < 0.10 in univariate analysis (surgical group, BMI, operative time, blood loss, diabetes) and clinically important variables forced into the model (age, FIGO stage) were entered into a multivariate binary logistic regression model. To isolate the independent effect of the surgical technique itself, operative time and blood loss—potential mediators—were excluded from the final model. The results (Table 7 and Figure 3, 4) showed that after adjusting for age, BMI, diabetes, and FIGO stage, undergoing systematic PLND remained an independent risk factor for postoperative complications, associated with a 4.732-fold increased risk compared to undergoing SLNB. Additionally, higher BMI was an independent predictor of complications.

**Table 7:** Multivariate logistic regression analysis of independent factors for postoperative complications.

Factor	*B*	SE	Wald	*P*-value	Adjusted OR (aOR)	95% CI
Surgical group (PLND vs. SLNB)	1.554	0.432	12.938	0.02	4.732	2.029–11.034
BMI (per 1 kg/m^2^ increase)	0.148	0.063	5.515	0.019	1.160	1.025–1.313
Diabetes mellitus (Yes vs. No)	0.791	0.488	2.629	0.105	2.205	0.847–5.742
Age (per 5-year increase)	−0.021	0.111	0.036	0.21	0.979	0.788–1.217
FIGO Stage (II vs. I)	0.542	0.506	1.147	0.65	1.719	0.638–4.632
Constant	−6.887	1.764	15.241	<0.001	0.001	–

### 3.8 Sensitivity analysis using propensity score matching

Propensity score matching created a well-balanced cohort of 46 pairs (SLNB = 46, PLND = 46). In this matched sample, the SLNB group continued to demonstrate a significantly lower overall complication rate (15.2% vs. 43.5%, *p* = 0.003) and a lower incidence of lymphedema at 12 months (4.3% vs. 23.9%, *p* = 0.013). The results for operative time, blood loss, and hospital stay remained significantly favorable for the SLNB group (all *p* < 0.01). This sensitivity analysis supports the robustness of the primary findings.

## 4 Discussion

This prospective observational study systematically evaluated the clinical utility of SLNB versus systematic PLND in the surgical management of endometrial cancer, with a focus on perioperative outcomes, complication risks, and patient-reported quality of life (16). Our findings clearly demonstrate that the SLNB technique, while ensuring oncological safety, significantly reduces postoperative complication rates, shortens operative time and hospital stay, and improves patients’ short-term quality of life (17). This comprehensive assessment provides robust evidence-based support for SLNB as a rational alternative for surgical staging in patients with low- to intermediate-risk endometrial cancer, offering important guidance for clinical practice and reflecting the trend toward precision and individualized therapy in gynecologic oncology surgery.

Regarding surgical and pathological outcomes, SLNB demonstrated high mapping success and bilateral detection rates, confirming its technical reliability and stability (18). Operative time, intraoperative blood loss, and postoperative hospital stay were significantly more favorable in the SLNB group compared to the systematic PLND group, highlighting its minimally invasive nature and superior procedural efficiency. This advantage likely stems from the precise, targeted removal of lymphatic tissue (19). Furthermore, although the overall node-positive rate did not differ significantly between groups, SLNB combined with pathological ultrastaging markedly improved the detection of micrometastases and isolated tumor cells. This indicates a unique diagnostic value of SLNB in identifying cases with low-volume metastatic disease, which may provide a more precise pathological basis for formulating subsequent adjuvant therapy strategies, thereby helping to avoid both under- and over-treatment.

Analysis of postoperative complications revealed that the overall complication rate and the proportion of severe complications were significantly lower in the SLNB group compared to the systematic PLND group, with a particularly notable difference in the risk of lower-limb lymphedema (20). This result underscores the substantial advantage of SLNB in reducing surgical trauma and associated morbidities, which is crucial for improving patients’ long-term quality of life. The underlying mechanism may involve the avoidance of extensive dissection of non-draining lymphatic basins, thereby minimizing disruption of lymphatic drainage, reducing secondary inflammatory responses, and lowering the risk of injury to local neurovascular structures (21). This finding aligns with results from several prospective studies, further corroborating the clinical value of SLNB in mitigating postoperative complications and providing compelling safety evidence to support its wider adoption.

In terms of patient-reported outcomes, the SLNB group showed superior scores in global health status and several functional dimensions during the early postoperative period compared to the PLND group, indicating a more comprehensive recovery of physical and psychological function (22). The improvement in quality of life is likely associated with reduced postoperative pain, fewer complications, and earlier recovery of mobility. It reflects the positive impact of avoiding unnecessary lymphadenectomy on overall patient rehabilitation, an effect manifested not only physiologically but also in psychological and social domains (23). This result reinforces the importance of incorporating patient-centered outcome measures into surgical evaluations, suggesting that the multidimensional impact of surgery on a patient’s quality of life must be carefully considered alongside the pursuit of oncological radicality.

Analysis of ultrastaging pathology revealed that SLNB, aided by immunohistochemistry, can identify low-volume metastases often missed by conventional staining, thereby optimizing the sensitivity of nodal assessment and improving the accuracy of disease staging (24). Although the exact prognostic significance of such findings remains debated, they provide a pathological foundation for further research into molecular biomarkers and individualized treatment strategies, also offering a new perspective for understanding the biology of tumor metastasis (25). The application of ultrastaging represents a more refined approach to disease staging, which may guide more precise postoperative therapeutic decisions in the future, propelling endometrial cancer management toward greater individualization and precision.

Multivariate regression analysis confirmed the surgical approach as an independent factor influencing postoperative complications, with SLNB significantly reducing the risk. This finding remained statistically significant after adjusting for multiple potential confounders (26). Concurrently, body mass index (BMI) was also identified as an independent predictor of complications, highlighting the importance of obesity and surgical complexity in perioperative management. These findings provide a basis for identifying high-risk patient populations and developing targeted preventive measures (27). The results emphasize the need in clinical practice to integrate patient characteristics with procedural factors to optimize treatment safety and achieve the best balance between therapeutic efficacy and patient well-being.

Guided by international consensus, SLNB is increasingly established as the standard of care for nodal assessment in early-stage endometrial cancer, supplanting routine systematic lymphadenectomy. In our institution, SLNB constitutes the primary nodal staging strategy. However, within the real-world clinical context during the study period, systematic PLND was still performed based on specific clinical scenarios, surgeon judgment, or patient preference after comprehensive counseling. This study, therefore, captures a comparative snapshot during a period of protocol evolution. Its contribution lies in providing prospective, detailed evidence on patient-centered outcomes that are critical for informed surgical choice but less frequently captured in trials focused on oncologic safety. By systematically employing standardized complication grading (Clavien-Dindo), a defined lymphedema assessment, and validated quality-of-life instruments (EORTC QLQ-C30), we quantify the significant perioperative and recovery benefits associated with SLNB. These findings reinforce the paradigm shift toward less morbid staging by delineating its tangible impact on reducing surgical trauma, severe complications, lymphedema risk, and accelerating quality-of-life recovery. The significant quality-of-life advantage observed in the SLNB group at 3 and 6 months had diminished by the 12-month assessment. This pattern likely reflects the natural trajectory of postoperative recovery. The more extensive surgical trauma associated with PLND results in a slower and more protracted recovery, manifesting as a larger QoL deficit in the short-to-medium term. Over a longer period (12 months), most patients, even those undergoing PLND, eventually achieve a functional and psychosocial baseline closer to their preoperative state or to that of the SLNB group, leading to a convergence of scores. This underscores that the primary benefit of a less invasive approach like SLNB is the acceleration of recovery and reduction of morbidity in the critical first year after surgery.

This study has several limitations. First, this study has the inherent limitations of a retrospective, non-randomized design. Group allocation was based on clinical decision-making and patient preference at the time of surgery, which introduces the potential for selection bias and limits causal inference, despite balanced baseline characteristics in our cohort. Although we adjusted for key patient and disease factors in multivariate analysis, residual confounding from unmeasured or imperfectly measured variables (e.g., detailed comorbidity burden, uterine size, or very subtle variations in surgical technique among the senior surgeons) cannot be entirely ruled out. Therefore, the results should be interpreted as demonstrating strong associations rather than definitive causation. Therefore, the results should be interpreted as demonstrating strong associations rather than definitive causation. Second, the single-center design and relatively limited sample size may constrain the generalizability of the findings, necessitating caution particularly in the interpretation of certain subgroup analyses. Future studies with larger samples are needed to validate these observations. Furthermore, this analysis was designed and powered for perioperative and quality-of-life endpoints. The follow-up duration (minimum 12 months) is insufficient to evaluate oncologic outcomes such as recurrence and survival. A separate, planned analysis with extended follow-up is ongoing to address these critical long-term endpoints. The absence of oncologic outcome data in the present report must be considered when interpreting the findings. Future research should validate our conclusions through multicenter, large-sample randomized controlled trials, explore the impact of SLNB on long-term oncological endpoints, and investigate potential differential treatment effects across various patient subgroups. Third, the assessment of lymphedema, while combining clinical measurement and patient report, has limitations. Our primary clinical definition relied on circumferential measurement at one or two sites. Although this aligns with common practice, it is less sensitive than volumetric assessment (e.g., perometry) or more complex multi-point criteria. Furthermore, inter-observer variability in tape measurement, though mitigated by using trained personnel and averaging, cannot be eliminated. The lack of a pre-operative baseline limb measurement is another constraint. Future studies would benefit from incorporating more objective volumetric tools and validated lymphedema-specific patient-reported outcome measures. Additionally, patient-reported lymphedema symptoms were captured via general inquiry rather than a validated, lymphedema-specific instrument such as the Lymphedema Functioning, Disability and Health questionnaire (Lymph-ICF) or the Gynecologic Cancer Lymphedema Questionnaire (GCLQ). The use of such tools in future studies would provide more precise and comparable data on the symptomatic burden. It is important to note that none of the patients in this cohort received postoperative external beam radiation therapy (EBRT), as adjuvant management for this low-to-intermediate risk population was limited to observation or vaginal brachytherapy based on final pathology. Therefore, the observed difference in lymphedema incidence is attributable to the surgical approach rather than confounding by adjuvant radiotherapy.

In conclusion, SLNB, as a less invasive surgical approach with a lower risk of complications, demonstrates considerable promise and value for adoption in patients with low- to intermediate-risk endometrial cancer. While preserving the accuracy of nodal assessment, it significantly improves perioperative outcomes and patient quality of life, reflecting the modern surgical trend toward precision and humanization. Our findings support the adoption of SLNB as the preferred nodal assessment strategy for eligible endometrial cancer patients in centers with proven technical expertise, aiming to maximize the surgical experience and postoperative quality of life for patients without compromising oncological safety.

## Data Availability

The raw data supporting the conclusions of this article will be made available by the authors, without undue reservation.
